# Real-Time PPG-Based Biometric Identification: Advancing Security with 2D Gram Matrices and Deep Learning Models

**DOI:** 10.3390/s25010040

**Published:** 2024-12-25

**Authors:** Ali Cherry, Aya Nasser, Wassim Salameh, Mohamad Abou Ali, Mohamad Hajj-Hassan

**Affiliations:** 1 Department of Biomedical Engineering, Lebanese International University, Beirut P.O. Box 146404, Lebanon; 31930594@students.liu.edu.lb (A.N.); mohamad.abouali01@liu.edu.lb (M.A.A.); 2 Department of Biomedical Engineering, International University of Beirut, Beirut P.O. Box 146404, Lebanon; 3 Department of Mechanical Engineering, Lebanese International University, Beirut P.O. Box 146404, Lebanon; wassim.salameh@liu.edu.lb

**Keywords:** photoplethysmography (PPG) signals, liveness detection, biometric security, two-dimensional format, Gram matrix conversion, deep learning, classification, real-time predictions

## Abstract

The integration of liveness detection into biometric systems is crucial for countering spoofing attacks and enhancing security. This study investigates the efficacy of photoplethysmography (PPG) signals, which offer distinct advantages over traditional biometric techniques. PPG signals are non-invasive, inherently contain liveness information that is highly resistant to spoofing, and are cost-efficient, making them a superior alternative for biometric authentication. A comprehensive protocol was established to collect PPG signals from 40 subjects using a custom-built acquisition system. These signals were then transformed into two-dimensional representations through the Gram matrix conversion technique. To analyze and authenticate users, we employed an EfficientNetV2 B0 model integrated with a Long Short-Term Memory (LSTM) network, achieving a remarkable 99% accuracy on the test set. Additionally, the model demonstrated outstanding precision, recall, and F1 scores. The refined model was further validated in real-time identification scenarios, underscoring its effectiveness and robustness for next-generation biometric recognition systems.

## 1. Introduction

**Figure 1:**
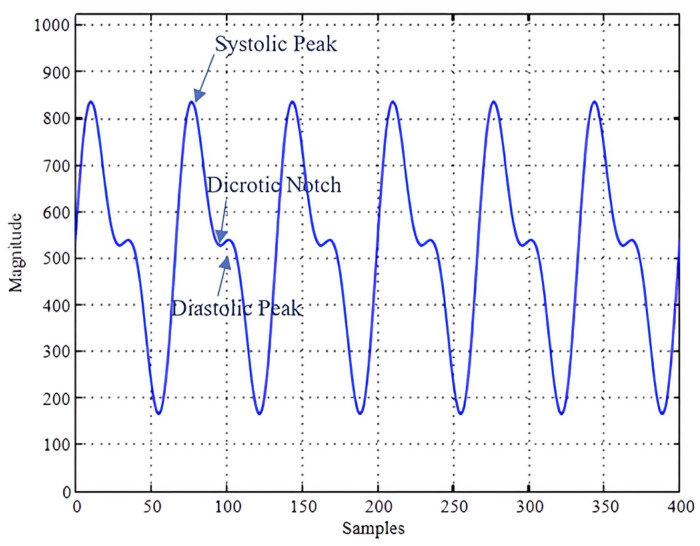
Features of the photoplethysmography (PPG) signal [4].

In today’s world, where technology and social media make various types of data vulnerable to unauthorized access through hacking and other methods, biometric identification has become increasingly crucial. Biometric systems, including facial recognition, fingerprint recognition, and iris recognition, offer significant advantages. They are uniquely tied to individual users—unlike passwords, which can be used without permission—and provide convenience as they require no memorization or physical possession. Additionally, these systems are highly resistant to fraud [1]. But still, these mentioned biometric identification techniques have not achieved a very high level of security; thus, the PPG signal is one of the preferred methods for identification. Photoplethysmography (PPG) is difficult to steal or replicate, which has the advantages of inherent anti-spoofing and liveness detection, and it can be conveniently recorded with just a combination of light-emitting diodes and photodiodes (PDs) from any part of the body and is, thus, very cost-effective compared to other biometric traits [2]. The PPG signal has a unique form that varies between individuals, which makes it useful for biometric identification after extracting its features. Its waveform is characterized by peaks and troughs aligning with each heartbeat. As shown in Figure 1, the PPG signal includes systolic peak/amplitude, which represents the maximum blood flow during a cardiac cycle; diastolic peak/amplitude, which represents the minimum blood flow; dicrotic notch amplitude, which represents a small dip in the PPG signal that occurs at the end of systole due to the closure of the aortic valve [3].

Exploring the potential of artificial intelligence for biometric identification, the adaptability of the PPG signal takes center stage. Whether represented in its innate 1D structure or subjected to a transformative shift into a 2D representation like a spectrogram, Gram matrix, or scalogram, the PPG signal unfolds as a rich and dynamic source of information.

Deep learning, a branch of machine learning, has garnered considerable interest for its capacity to identify intricate patterns and representations from extensive datasets [5].

Deep learning models, including convolutional neural networks (CNNs) and recurrent neural networks (RNNs), are highly effective at extracting significant features and delivering precise predictions [6]. Deep learning models excel at identifying complex patterns and adapting to individual variations, which improves their accuracy and reliability in biometric identification. PPG-based authentication systems further benefit from the capability to provide continuous and passive verification, facilitating seamless user authentication in various scenarios [7]. However, integrating PPG with deep learning presents challenges, such as variations in PPG signals due to skin tone, motion artifacts, and environmental factors, which can impact system performance. Additionally, the effective collection and preprocessing of high-quality PPG data are crucial for developing robust deep learning models. Addressing these challenges involves meticulous data handling, sophisticated preprocessing techniques, and careful model optimization.

This paper seeks to enhance the performance of existing shortening algorithms for PPG signal recognition in 2D representation. We propose innovative solutions to overcome accuracy limitations, reduce training requirements, optimize computational efficiency, and address overfitting issues while considering practical constraints such as dataset size and computational resources. To achieve these goals, we implemented a comprehensive protocol for collecting PPG signals from 40 subjects, applied filtration and augmentation techniques during preprocessing, and converted the data into a two-dimensional format using Gram matrices. Our study culminates in the classification of PPG signals for real-time prediction systems, aiming to improve the effectiveness and efficiency of biometric recognition applications.

Our work makes significant contributions to the field of biometric authentication by exploring the efficacy of photoplethysmography (PPG) signals in combination with advanced deep learning techniques. The key contributions of our research are as follows:

1. Signal Acquisition and Data Processing: We developed a comprehensive protocol for collecting PPG signals using a custom-built, low-cost acquisition system compatible with widely available microcontroller platforms. This system is designed to be efficient, reliable, and user-friendly, featuring straightforward programming interfaces that make it accessible even to non-expert developers. The collected signals were processed into two-dimensional representations using the Gram matrix conversion technique, which enhances the analysis of physiological data.
2. Advanced Biometric Authentication Model: We employed a hybrid model combining EfficientNetV2 B0 with a Long Short-Term Memory (LSTM) network for user authentication. This approach achieved an impressive 99% accuracy on the test set, demonstrating its robustness and effectiveness in identifying individuals.
3. Real-Time Validation and Performance Metrics: Extensive evaluations in real-time identification scenarios highlighted the model’s high precision, recall, and F1 scores. These metrics underscore the practical implications of our work, demonstrating PPG’s reliability for continuous biometric authentication in various environments.

Overall, this research addresses key challenges in biometric authentication by leveraging the distinct advantages of PPG signals and advanced deep learning methodologies, paving the way for enhanced security and reliability in biometric systems.

## 2. Related Works

Biometric authentication utilizing PPG signals has garnered considerable interest in recent years. These signals, which reflect volumetric changes in blood vessels, offer distinct physiological data useful for user identification and authentication. Deep learning methods, such as convolutional neural networks (CNNs), recurrent neural networks (RNNs), and transformer-based models, have been employed to extract features and identify patterns within PPG signals [8]. There are some previous works of 2-D representation for several biological signals like ECG, EMG, EEG, and PPG for biometric identification, which will allow us to analyze and prove the discriminability of PPG signals as biometric traits.

Li Zhang et al. developed a biometric authentication system utilizing PPG signals combined with both deep learning (DL) and traditional machine learning techniques. Their research showcased the efficacy of deep learning models, particularly convolutional neural networks (CNNs), in accurately identifying individuals based on PPG signals obtained from the fingertip [9]. The study emphasized the significance of feature extraction and preprocessing in enhancing system performance. It proposed a deep learning framework that merges convolutional and recurrent neural networks to extract and classify features from PPG signals. The results highlighted the method’s effectiveness, demonstrating high accuracy in biometric identification tasks.

Hanilci et al. proposed a biometric identification method using ECG signals, employing a parallel 2-D convolutional neural network (CNN) architecture. This approach was designed to minimize computational time while maintaining a high level of accuracy. The method achieved an accuracy of 88.57%, demonstrating its effectiveness in efficiently processing ECG data for biometric identification [10]. By leveraging parallel processing within the 2-D CNN framework, their approach not only enhanced the speed of computations but also ensured robust performance in accurately identifying individuals based on their ECG signals. This advancement represents a significant step forward in the optimization of biometric systems utilizing ECG data.

M. Kim et al. studied the possibility of recognition using a 2D ECG ensemble of deep convolution neural networks; this method leverages deep CNNs to achieve enhanced overall performance by significantly improving reliability, accuracy, and adaptability in user identification tasks based on ECG data [11]. However, the adoption of deep CNNs introduces increased computational complexity due to the sophisticated nature of the neural networks involved. This method achieves a recognition performance percentage of 97.2%.

J. Kim et al. proposed a two-step biometric method using EMG signals based on a convolution neural network–Long Short-Term Memory (CNN-LSTM) network. The findings demonstrated a 99.17% single biometric performance and an 83.91% gesture recognition performance [12]. Additionally, it was found that data fusion lowered the false acceptance rate (FAR) by 64.7%.

D. Labati et al. suggested a method that can be used for both identification and identity verification. A PPG signal with a set time length of t seconds is sent into it. It computes the matching phase using machine learning classifiers, extracts features from various transformations of the PPG signal spectrogram, and conducts biometric recognitions without the need to extract fiducial points. This method achieved an accuracy of 99% [13].

M. Svetlakov et al. suggested employing Hilbert spectrograms of the data as a subject-independent learning mechanism for EEG-based biometrics. The architecture was tested, and an Equal Error Rate (EER) of 14.63% was attained. This algorithm provided an accuracy of 64.75% [14].

Bengie L. Ortiz et al. proposed leveraging PPG signals as a biometric indicator by employing an end-to-end learning approach combined with an XgBoost classifier. In the process of acquiring PPG signals, they carefully considered unique physiological factors to select relevant features. For classification, the XgBoost algorithm was utilized, which demonstrated notable performance. The approach achieved an accuracy (ACC) of 97.36%. Additionally, their method resulted in an area under the curve (AUC) of 83 and an average accuracy of 78.2% [15]. These metrics highlight the effectiveness of their method in utilizing PPG signals for biometric authentication, reflecting strong performance in distinguishing between individuals based on physiological data.

M. Ibrahim et al. presented a method for utilizing PPG cardiac signals to confirm people’s identities. Using the scalogram approach, these preprocessed PPG signals were converted into a 2-D image that graphically depicts the time-varying frequency content of several PPG signals from the same person. Next, the features fusion approach is developed by combining features from the hybrid convolution vision transformer (CVT) and convolutional mixer (ConvMixer), which is known as the CVT-ConvMixer classifier. This approach achieves an accuracy of 95% [16].

Caiyu Wu et al. introduced an advanced deep learning framework for biometric identification utilizing photoplethysmography (PPG) signals, which are frequently gathered in everyday settings. Addressing the challenges that current PPG-based systems face due to motion artifacts and varying physiological conditions, the authors developed a novel deep convolutional neural network (CNN) that incorporates Gram matrix techniques to convert time-series PPG data into two-dimensional representations. This transformation enhances the capture of temporal dependencies, significantly boosting identification accuracy. Their model, evaluated on the TROIKA dataset under ambulatory conditions, demonstrated notable performance gains, with accuracy improving from 69.5% to 92.4% in multi-class classification [17].

These studies explore the application of biological signals such as ECG, EMG, EEG, and PPG for biometric recognition by transforming them into two-dimensional representations. This approach is substantiated by evidence indicating that these signals provide enhanced security and superior accuracy compared to alternative methods. Additionally, the integration of PPG with deep learning (DL) techniques has revealed significant potential for biometric authentication. Deep learning models, when applied to PPG signals from diverse body parts or through remote sensing, have demonstrated effective individual identification capabilities. Building upon these foundational advancements, our research further enhances the field of biometric identification by advancing existing methodologies.

To offer a comprehensive assessment of recent advancements in biometric recognition, we present a comparison table (Table 1) highlighting the advantages and limitations of previous studies. This table consolidates key factors such as performance metrics, computational requirements, and practical challenges associated with various biometric signal modalities, including ECG, EMG, EEG, and PPG.

## 3. Materials and Methods

In this section, we thoroughly analyze our experimental framework, meticulously designed to harness PPG signals for biometric recognition. Our study employs data collected from 40 subjects using the acquisition system we developed. This work builds upon our previous research [18], which involved a comparative study using an online PPG dataset sourced from the Real World PPG Dataset [19]. In that study, we evaluated three different signal conversion techniques and trained them on five distinct models to identify the top-performing model and the most effective 2D conversion technique. The results indicated that the most effective model was EfficientNetV2 B0 integrated with LSTM, utilizing the Gram matrix as the 2D conversion technique. Consequently, this model and conversion technique will be employed in the current study.

We begin with a detailed examination of the acquisition system, addressing the challenges of capturing high-quality PPG signals. Next, we outline the critical stages of our methodology, including signal preprocessing, conversion to a 2D format, and the application of data augmentation techniques. The final stage involves classifying PPG signals within a real-time prediction system, enabling us to test our hypotheses and evaluate the robustness of our approach.

### 3.1. System Architecture

**Figure 2:**
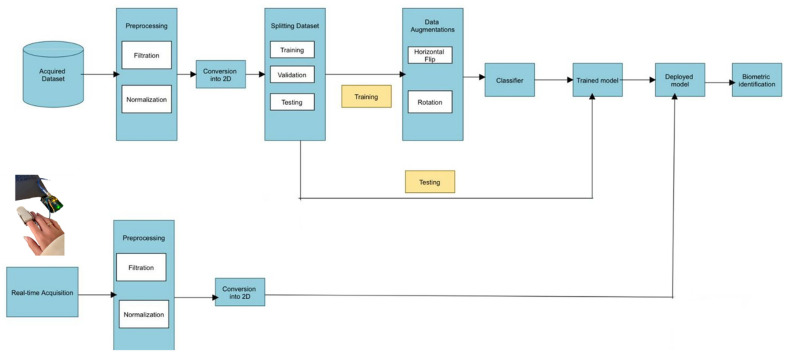
Block diagram of the developed system.

The system’s architecture diagram is presented in Figure 2 illustrates the detailed architecture and connections within the proposed system. This diagram enhances comprehension of the system’s operation and highlights the smooth integration of various components. The strategic layout of blocks and the directional flow of information outlined in the diagram offer a clear roadmap, helping readers to navigate the complexities of the research framework.

The initial phase of our system involves acquiring PPG data using our setup, followed by preprocessing steps that include normalization and filtration. After preprocessing, the dataset is converted into a 2D format and divided into training, validation, and testing subsets. The training data are then augmented and processed using deep learning techniques. Finally, the model’s performance is assessed using the test dataset.

In real-time acquisition, PPG signals are collected with a SpO2 sensor typically mounted on the finger. The signals are then preprocessed through normalization, filtration, and rescaling. The processed data are converted into a 2D format and input into a pre-trained model, which analyzes it to identify the user accurately.

### 3.2. Data Acquisition

The data acquisition process involves capturing PPG signals through a well-defined protocol and experimental setup. This section includes a detailed description of the procedures used to collect the signals, the equipment employed, and the conditions under which the data were obtained. An overview of the acquired dataset will also be provided, highlighting the specifics and characteristics of the collected PPG signals.

#### 3.2.1. Acquisition Protocol

To guarantee the reliable acquisition of PPG signals, a comprehensive protocol is established, detailing the data collection process with precision. This protocol includes specific guidelines for sensor placement, environmental conditions, and preparatory procedures. Adhering to a robust protocol ensures consistency and accuracy, enabling the collection of high-quality PPG signals for precise biometric identification. The PPG sensor should be correctly positioned to avoid interference from external light sources, and it should be secured with additional measures, such as taping, to reduce noise from contact with the soldered area. The entire setup should be shielded to protect it from environmental factors. Users should maintain a relaxed and stable posture, free from sources of noise and disturbances. A brief acclimatization period is recommended before beginning the recording process, which should last a minimum of 10 min. Additionally, ensuring that the fingertips are warm is crucial, as cold extremities can lead to reduced perfusion and negatively impact the quality of the PPG signal.

#### 3.2.2. Experimental Setup

**Figure 3:**
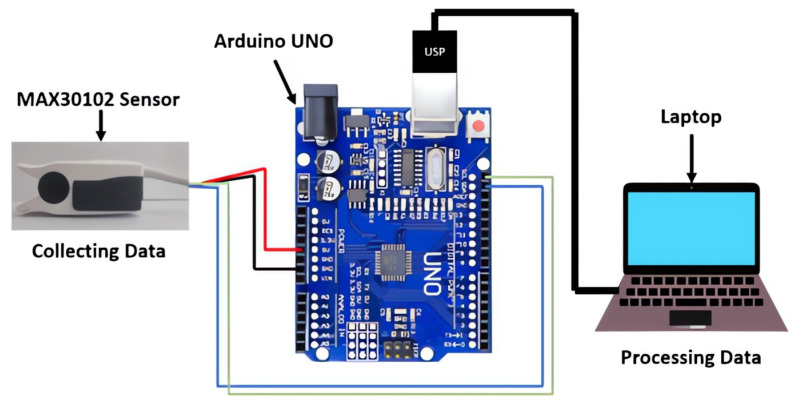
Complete acquisition system design.

Our system is a biometric identification platform that utilizes photoplethysmography (PPG) signals for precise identification. It consists of several key components designed to capture and process these signals effectively. The main components include the MAX30102 sensor, sourced from Analog Devices, Inc., Norwood, MA, USA; an Arduino UNO microcontroller, sourced from Arduino S.r.l., Turin, Italy; and an SpO2 sensor cover from a Nellcor pulse oximeter, sourced from Medtronic, Minneapolis, MN, USA. Figure 3 shows the fully assembled prototype of the system.

**Figure 4:**
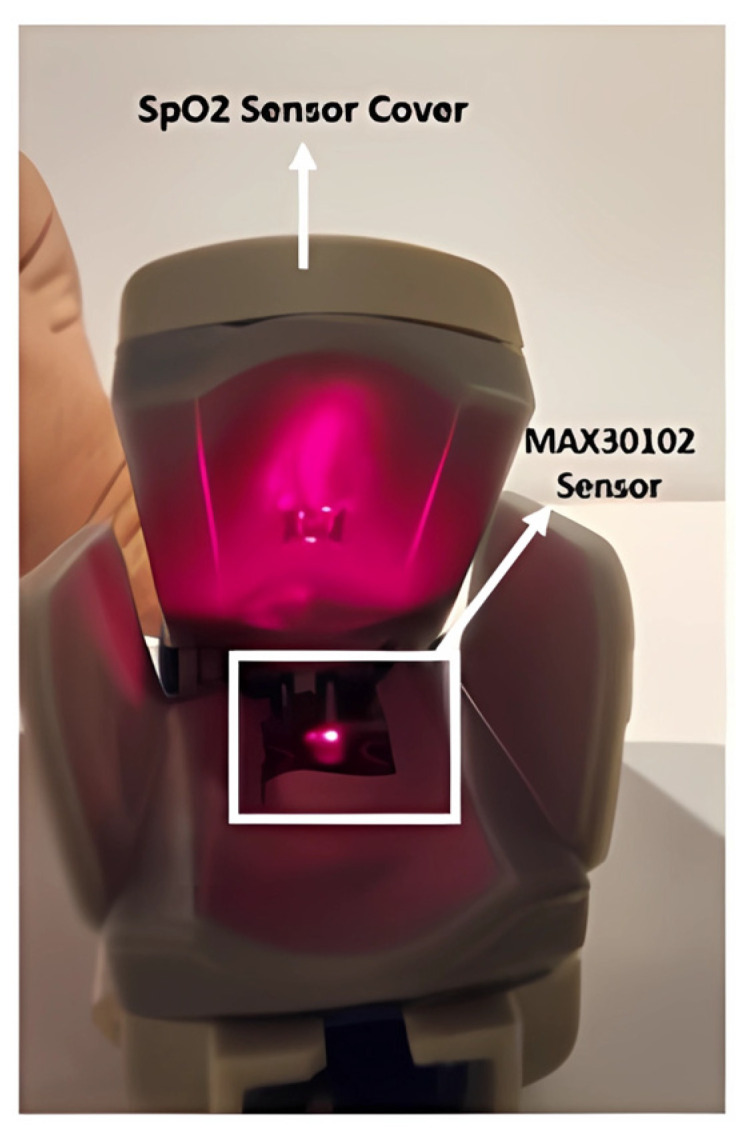
Sensor implementation.

During the implementation phase, the initial hardware component chosen was a Nellcor SpO2 sensor. The infrared light source and detector, along with their associated wiring, were carefully removed from the sensor. These components were then replaced with the Arduino-compatible MAX30102 sensor, which was integrated to closely mimic the original SpO2 sensor setup. The MAX30102 sensor utilizes specific wavelengths of light—red at approximately 660 nm and infrared (IR) at around 940 nm—for accurate measurement of biometric signals. Integrated within the sensor is a highly efficient analog-to-digital converter (ADC) capable of converting the analog signals from the photodetectors into digital data with 18-bit resolution. This ADC operates at a sampling rate of up to 1 kHz, enhancing the fidelity of the captured biometric information. Figure 4 demonstrates how the MAX30102 was installed within the SpO2 cover, illustrating its operational setup.

The sensor was subsequently connected to the Arduino board using four wires, each linked to the appropriate pins to facilitate data reading from the sensor.

#### 3.2.3. Acquired Dataset

PPG data signals were collected from 40 healthy individuals aged 14 to 70, with each subject providing data individually. The acquisition protocol was rigorously adhered to, and the environment was meticulously controlled to minimize noise interference. For each participant, 80 PPG signals were recorded, with each signal comprising 300 samples. Consequently, the total acquired dataset consists of approximately 3200 PPG signals.

The data collected by the Max30102 sensor, interfaced with an Arduino, is efficiently stored in an Excel spreadsheet on a computer using the ArduSpreadsheet tool. This organized approach ensures easy access and efficient management of the data. To leverage the analytical capabilities of the collected data, we used MATLAB to transform the structured information from the Excel sheet into a clear signal format. This transformation not only provides better visualization of the data’s underlying patterns but also prepares the data for further preprocessing and analysis in our study of PPG signals for biometric identification.

**Figure 5:**
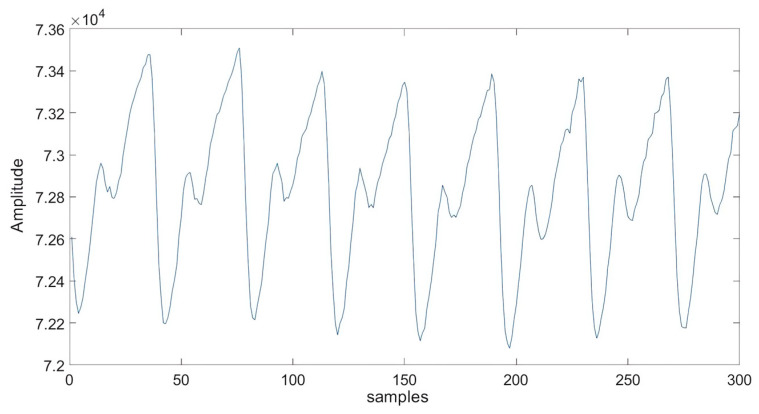
Unfiltered PPG signal acquired by our experimental system.

Figure 5 shows a PPG signal captured by our experimental system and visualized using MATLAB. The plot depicts discrete samples from the MAX30102 sensor’s analog-to-digital converter, with the y-axis representing light intensity reflected from the tissue, ranging from 0 to 262,143. The x-axis denotes the number of samples collected.

### 3.3. Preprocessing

Preprocessing is a crucial stage in signal analysis. It enhances accuracy and reliability by ensuring data consistency and improving the clarity of the data for algorithms [20].

Filtration and normalization are vital for improving and refining data quality before it is further analyzed. Filtration techniques are used to reduce or remove unwanted noise, preserving the core information and integrity of the signal. This step is essential for enhancing the signal-to-noise ratio and ensuring the accuracy of the analysis that follows. Conversely, normalization techniques standardize the amplitude and range of the signal, providing consistency and comparability across different datasets. By reducing variations and aligning signal characteristics, normalization helps create a more dependable and robust basis for subsequent processing and interpretation. Together, these preprocessing methods establish a solid foundation for deriving meaningful insights from the raw data.

#### 3.3.1. Filtration Technique

**Figure 6:**
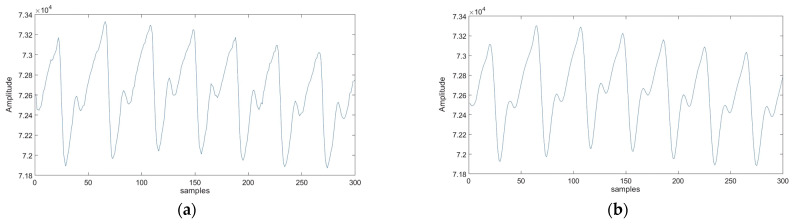
Visual representation of preprocessed PPG signal: (**a**) original PPG signal; (**b**) filtered PPG signal using the Savitsky-Golay filter.

The signals we acquire, as illustrated in Figure 6a, are often impacted by additive noise, which can compromise signal integrity and adversely affect feature extraction and overall system performance. To address these issues and ensure robust subsequent analyses, we implement a crucial preprocessing step using the “Savitsky-Golay” filter. This filter, specifically designed for digital data, enhances accuracy while preserving the overall trend of the signal. It achieves this by applying convolution, fitting low-degree polynomials to successive subsets of adjacent data points through linear least squares.

By employing the Savitsky-Golay filter, as shown in Figure 6b, we effectively reduce various noise effects, thereby laying a solid foundation for precise feature extraction and a deeper understanding of the PPG signal’s behavior. This polynomial fitting process reduces high-frequency noise while maintaining the essential features of the signal. Unlike traditional filters, which may remove important slow-varying components that provide valuable physiological insights, the Savitzky-Golay filter preserves both fast and slow-varying components. This ensures that no critical information is lost during signal processing.

When data points are evenly spaced, an analytical solution to the least-squares equations can be derived. This solution, represented by a set of “convolution coefficients”, is applied uniformly to all data subsets, resulting in a smoothed signal estimation or its derivatives at the center of each subset [21].

#### 3.3.2. Normalization

Normalization can be achieved through various methods, each tailored to specific purposes, but differences in range and scale can complicate direct comparisons. To address this, we apply normalization to align both signals within a common, predefined range, ensuring consistency and facilitating accurate comparison.

**Figure 7:**
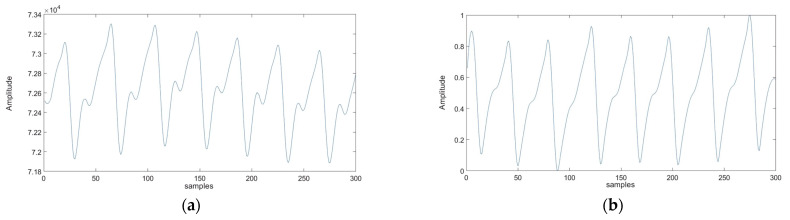
Visual representation of preprocessed PPG signal: (**a**) non-normalized PPG signal; (**b**) normalized PPG signal.

Min–max normalization, also known as feature scaling, is described by Equation (1). In this method, we adjust the signals to be within a specific range, typically [0, 1] (see Figure 7). This process involves subtracting the minimum value from each data point and then dividing by the range (the difference between the maximum and minimum values). After normalization, both signals share the same statistical characteristics, enabling more meaningful and accurate comparisons.

(1) $$x^{\prime }=\frac{x-min(x)}{max\left(x\right)-min(x)}$$

This process facilitates visualizations, analyses, and comparisons by removing the effects of varying scales and ranges. It also reduces computational time during classification. By standardizing signals to a common scale, whether for graph plotting or statistical analysis, the robustness and interpretability of the data are improved. This ensures that patterns and trends can be more accurately identified and understood [22].

#### 3.3.3. Signal Transformation into Gram Matrix

Transforming a 1-D PPG signal into a 2-D Gram matrix is crucial for effective signal processing and analysis. This conversion yields a time–frequency representation that enhances feature extraction, leading to more accurate and robust pattern recognition. The 2-D Gram matrix not only visualizes the signal’s behavior but also provides a richer input for machine learning applications by encapsulating temporal correlations and revealing complex modulation patterns within the PPG signal.

The process of computing a Gram matrix involves the following steps:

1. Initialization: A cell array named “signals” is initialized to store the signal data. These signals are imported into MATLAB and converted into arrays, and each signal is stored as a row in the cell array.
2. Gram Matrix Calculation: For each pair of signals, $s_{j}$ and $s_{i}$, the Gram matrix entry $G_{ij}$ is computed based on Equation (2):

(2) $$G_{ij}=s_{i}.s_{j}=\sum_{K=1}^{N}s_{i,k}.s_{j,k}$$

Here, $s_{i}\;$ and $s_{j}\;$ represent the *i*-th and *j*-th signals, respectively, and N denotes the length of each signal. This computation captures the pairwise dot products between signals, which are essential for assessing their similarities.

The resulting Gram matrix  G is an M×N  matrix, where M is the number of signals.

3. Visualization: Once the Gram matrix is computed, it is visualized using the imagesc function in MATLAB. This visualization presents the matrix as an image, with brighter regions indicating higher similarity between signals. A color bar is included for reference.
4. Applications: The Gram matrix and its visualization play a crucial role in various applications, including pattern recognition, similarity assessment, and feature extraction. The visual representation provided by the Gram matrix enhances the interpretation and analysis of complex datasets by clearly illustrating signal relationships. This approach is particularly beneficial in fields like biomedical signal processing, audio analysis, and machine learning. Additionally, the versatility of the Gram matrix computation makes it applicable to biometric recognition systems, where it can be used to compare signals such as fingerprints, iris patterns, PPG signals, or voice recordings. This enhances the accuracy and security of biometric authentication technologies by improving identification and verification processes [23].

**Figure 8:**
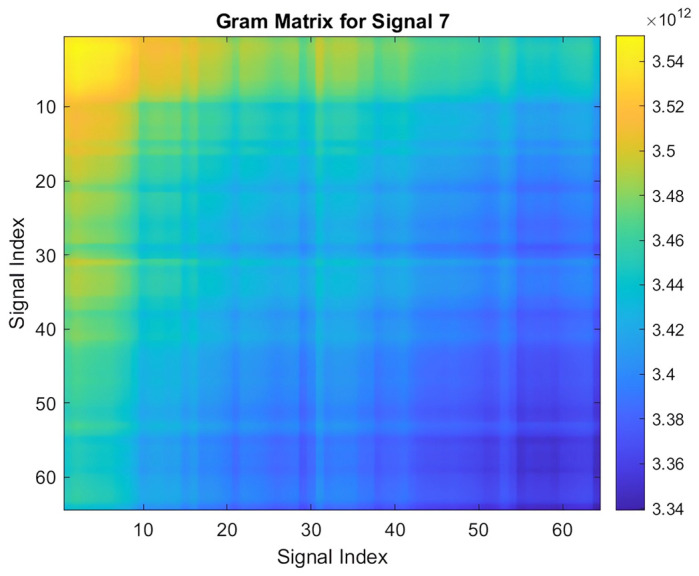
Gram matrix of an acquired PPG signal.

Applying this transformation yields a Gram matrix for each PPG signal, resembling the one depicted in Figure 8.

#### 3.3.4. Data Splitting

In machine learning, data splitting is commonly employed to prevent overfitting. Overfitting occurs when a model learns the training data too precisely, which hampers its ability to generalize to new, unseen data [24].

To mitigate this issue, the original dataset is usually divided into two or three subsets, facilitating effective model training and evaluation. The commonly used subsets are as follows:

- Training Set: This subset is utilized to train the model, allowing it to learn and optimize its parameters based on these data.
- Validation Set: Also known as the cross-validation or model validation set, this subset is used to adjust learning parameters and evaluate the model’s performance. It helps in assessing the model’s accuracy and is instrumental in model selection.
- Testing Set: This subset is used to assess the final model’s performance. It provides an evaluation of how well the model generalizes to unseen data by comparing its results with those from the training and validation sets.

To ensure sufficient data for training, datasets are often split using ratios such as 80–20 or 70–30 for training versus testing. The specific ratio may vary depending on the dataset size, but an 80–20 split is generally considered effective for smaller datasets [25].

Based on our acquired dataset of 40 healthy individuals aged between 14 and 70, we have a total of approximately 3200 PPG signals. Given the relatively small size of this dataset, we have divided it into two subsets: one for training the classifier, containing 2560 PPG signals (about 80% of the dataset), and one for testing, consisting of approximately 640 PPG signals (about 20%).

#### 3.3.5. Data Augmentation

During the data augmentation phase, we exclusively utilize the training set. This deliberate approach allows us to introduce controlled variations and enhance the diversity of our training data, which in turn helps the model generalize more effectively and learn patterns more robustly. By limiting data augmentation to the training set, we ensure that the instances used for model training are enriched, leading to a more robust training process. Data augmentation enhances classification accuracy by broadening and diversifying the dataset, thus mitigating overfitting to random noise. However, it is important to note that data augmentation strategies are inherently specific to the domain [26].

In the context of Gram matrix, applying a horizontal flip using Principal Component Analysis (PCA) and singular value (SV) perturbation involves altering the time–frequency representation [27].

PCA is a dimensionality reduction technique that identifies the principal components, or directions of maximum variance, within a dataset. By transforming the data into a new coordinate system defined by these principal components, PCA reduces the dimensionality while retaining the most important information. In the context of data augmentation, PCA can be used to generate variations by manipulating the principal components, introducing controlled transformations that simulate different aspects of the original data [27].

Singular value perturbation involves adding controlled variations to the singular values of a matrix. When used for data augmentation, this technique adjusts the singular values of the original data matrix, introducing controlled noise or variations. This approach generates diverse data instances while maintaining the core features of the original dataset.

**Figure 9:**
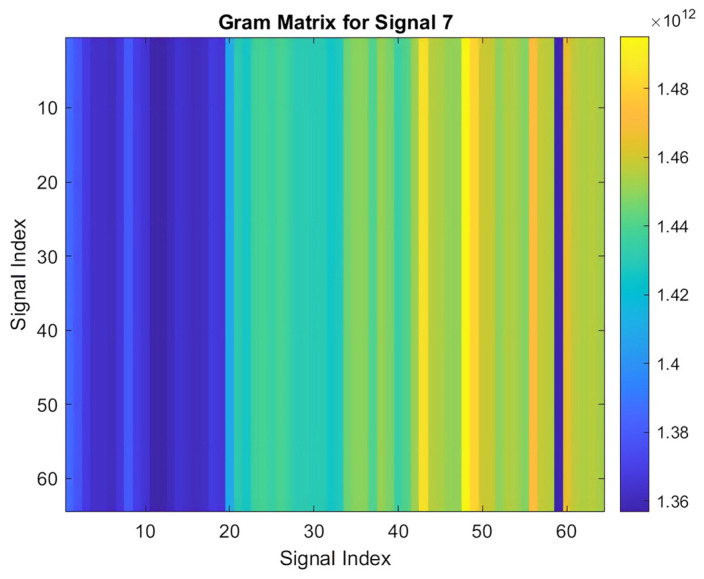
Gram matrix after applying horizontal flip augmentation.

In summary, PCA and singular value perturbation are crucial for data augmentation as they introduce controlled variations that enhance the diversity of the dataset. Figure 9 illustrates the Gram matrix generated after applying the PCA and singular value perturbation, including the horizontal flip technique.

**Figure 10:**
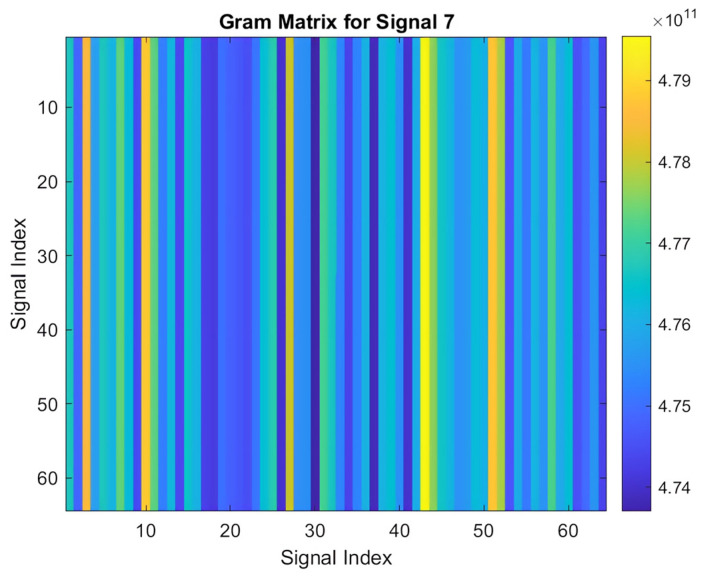
Gram matrix after applying rotation augmentation.

Another augmentation method employed involves rotating images within a specified range of up to 90 degrees, as depicted in Figure 10. During training, each image in the dataset can be randomly rotated within this range. This strategy introduces variations in image orientation, allowing the model to learn from a wider range of perspectives. By exposing the model to rotated images, it becomes better at recognizing and classifying objects. This technique helps reduce overfitting and enhances the model’s generalization.

Finally, the initial training subset of 2560 Gram matrices was augmented to a total of 28,562 using both previously mentioned augmentation techniques, significantly expanding the dataset for training the selected EfficientNetV2 B0 model, sourced from the Keras deep learning library.

### 3.4. Model Structure

**Figure 11:**
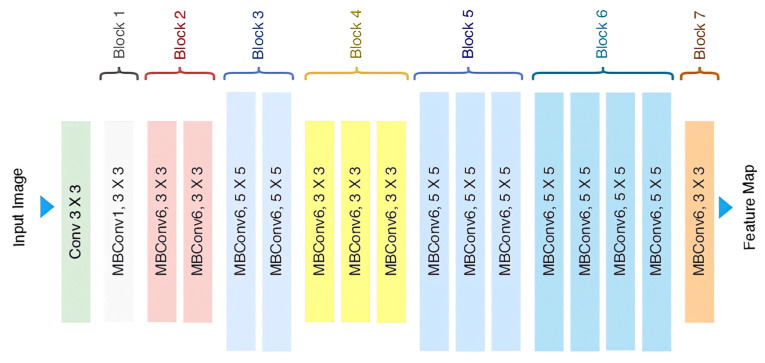
EfficientNetV2 B0 model architecture [28].

The EfficientNetV2 B0 model implemented in our study, as illustrated in Figure 11, typically consists of approximately 66 layers, including both convolutional and fully connected layers. These layers enable the network to extract hierarchical features from input images, facilitating efficient and effective image classification, object detection, and semantic segmentation.

Key characteristics of EfficientNetV2 B0 include:

Depth, Width, and Resolution: EfficientNet models are scaled based on three factors—depth, width, and resolution. The B0 variant serves as the baseline with moderate depth, width, and resolution.

Compound Scaling: EfficientNetV2 employs compound scaling to optimize model capacity and computational efficiency across different dimensions. Rather than uniformly scaling all dimensions, compound scaling evaluates the impact of each dimension on overall model efficiency, thereby maximizing performance while minimizing computational costs.

Stem Convolutional Layer: EfficientNetV2 starts with a stem convolutional layer calculated by Equation (3) that processes input images and extracts fundamental features. This layer typically involves a series of convolutional and pooling operations to downsample the input and increase the number of channels [29].

(3) $$y_{\left(i,j,k\right)}=\sum_{m=1}^{M}\sum_{n=1}^{N}\sum_{c=1}^{C}x\left(i+m,j+n,c\right).Q(m,n,c,k)+B(k)$$

where

- $y_{\left(i,j,k\right)}$ is the value of the output feature map at position (*i*, *j*) for k-th channel;
- $x\left(i+m,j+n,c\right)$ is the value of the input feature map at position (*i + m*, *j + n*) in c-th channel;
- Q(m,n,c,k) is the weight of the convolutional kernel at position (*m*, *n*) for the c-th input channel and k-th output channel;
- B(k) is the bias term for the k-th output channel;
- *M* and *N* are the dimensions of the kernel;
- *C* is the number of input channels.

Inverted Residual Blocks: The architecture utilizes inverted residual blocks calculated using Equation (4), which consist of depthwise separable convolutions followed by pointwise convolutions. This design reduces computational complexity while maintaining representational capacity, making it particularly effective for mobile and embedded devices [30].

(4) $$\;y=x+\sigma \left(x+W_{dw}\right)*W_{dw}$$

where

- x  is the input tensor.
- $W_{dw}\;$ is the weight matrix for the depthwise convolution.
- $W_{pw}\;$ is the weight matrix for the pointwise convolution.
- σ represents a non-linear activation function applied element-wise.

Squeeze-and-Excitation Blocks: EfficientNetV2 includes squeeze-and-excitation (SE) blocks, which will be calculated by Equation (5), that adaptively recalibrate channel-wise feature responses. SE blocks enhance the network’s representational power by modeling interdependencies between channels, thereby improving both accuracy and efficiency [30].

(5) $$Z_{squeeze}=\frac{1}{H\times W}\sum_{h=1}^{H}\sum_{w=1}^{W}X_{h,w}\;Z_{excite}=\sigma \;(W_{1}.\left(W_{2}.Z_{squeeze}+b_{1}\right)+b_{2}$$

where

- $Z_{squeeze}\;$ is the channel with global average pooling results.
- $Z_{excite}\;$ represents the excitation output.
- σ represents the sigmoid activation function.
- $W_{1}{\;,W}_{2},$ $b_{1}{\;,b}_{2}$ are the weights and biases of the excitation network.

Swish Activation Function: EfficientNetV2 frequently employs the Swish activation function, which can outperform traditional functions like ReLU in certain situations. Swish is a smooth, non-monotonic function that combines aspects of sigmoid and ReLU activations, potentially leading to better gradient flow and improved learning.

**Figure 12:**
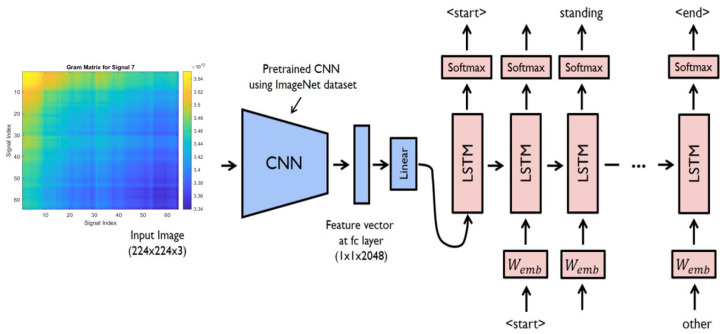
Deep learning CNN and LSTM [28].

Combining EfficientNetV2 B0 with a recurrent neural network (RNN), such as Long Short-Term Memory (LSTM), offers a robust solution for tasks involving sequential data or temporal dependencies. The architecture of this integrated model is illustrated in Figure 12.

**Figure 13:**
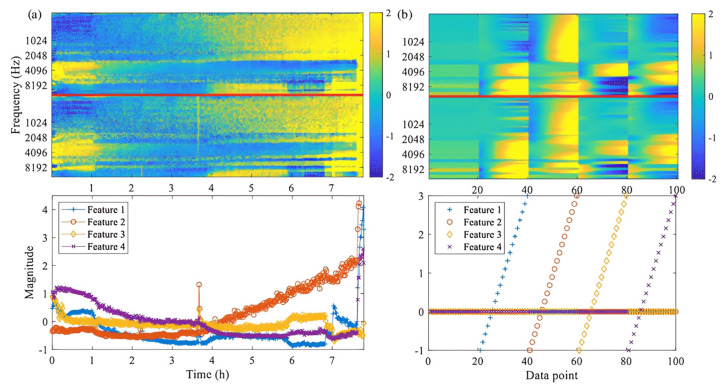
High-level feature extraction process using EfficientNetV2 B0. (**a**): Response of the high-level features of the trained autoencoder due to the changes in the magnitudes of inputs of the autoencoder. (**b**): Response of the outputs of the autoencoder due to the changes in the high-level feature values [28].

Feature Extraction with EfficientNetV2 B0: Initially, EfficientNetV2 B0 is used to extract high-level features from each frame in the sequence, as shown in Figure 13. EfficientNetV2 B0 acts as the feature extractor, converting raw image data into a rich representation that effectively captures spatial information.

Temporal Processing with LSTM: Once the features are extracted by EfficientNetV2 B0, you can pass them to the LSTM network for temporal processing. The LSTM network can analyze the sequence of features over time, capturing temporal dependencies and patterns in the data.

Integration and Classification: The output from the LSTM network can be aggregated across time steps to create a fixed-size representation of the entire sequence. This representation is then used as input for additional layers, such as fully connected layers, to perform classification or regression tasks.

Training and Optimization: The complete model, which includes both the EfficientNetV2 B0 feature extractor and the LSTM network, is trained end-to-end through backpropagation and optimization methods like stochastic gradient descent (SGD) or Adam. During training, the parameters of both the EfficientNetV2 B0 and the LSTM are updated concurrently to minimize the selected loss function.

### 3.5. Fine-Tuning

Fine-tuning is critical for optimizing the accuracy of deep learning models, particularly when using pre-trained architectures such as EfficientNetV2 B0 integrated with LSTM. In our study, we aimed to enhance model performance through an ablation study that systematically assessed the impact of four key hyperparameters: batch size, activation function, learning rate, and optimizer. Each of these parameters was varied across three distinct settings, enabling us to observe and analyze their effects on model performance. The results for each configuration revealed significant variations, highlighting how these hyperparameters influence the model’s learning and generalization capabilities. This iterative process not only underscored the model’s sensitivity to these parameters but also enabled us to identify the optimal configuration, ensuring the selection of the best components to achieve superior accuracy. Table 2 presents the documented variations for each hyperparameter across the different settings.

### 3.6. Real-Time Application

In this section, we outline the approach employed in PyCharm, version 2023.2, to develop a real-time biometric identification system. The implementation of the real-time system starts with the program fetching data from the Arduino device, which is connected to the computer through PyCharm software. After establishing this connection, the system allows a 25 s stabilization period to ensure the signal is steady before capturing and preprocessing the PPG signal. During preprocessing, several steps are carried out, including normalization, filtering, and converting the signal into a 2D image using the Gram matrix method. The processed data are visualized through plots, and the predicted individual’s name is shown using a GUI (Graphical User Interface) built with Tkinter, version 3.11.1.

**Figure 14:**
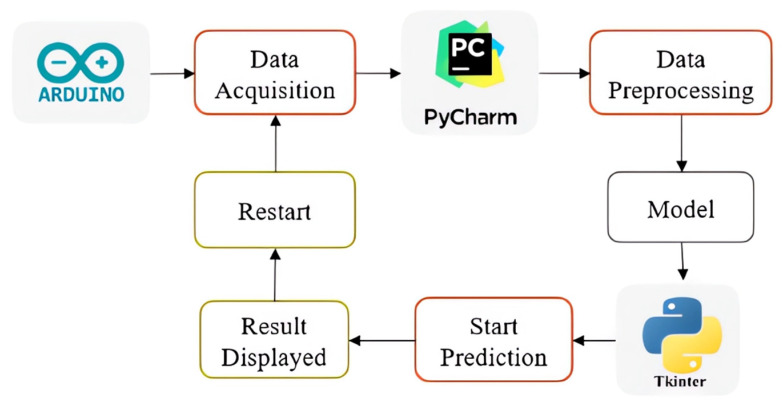
Methodology of real-time implementation, detailing system architecture and data flow.

The GUI includes buttons to start the prediction process, allowing results to be displayed in a separate window. It also features a restart button to enable the re-running of the entire process. A graphical block diagram, presented in Figure 14, visually represents the implementation process. This diagram provides a comprehensive overview of the system architecture, detailing the data flow and key processing stages, including data retrieval, preprocessing, prediction, and GUI display.

## 4. Results

### 4.1. Performance Evaluation

**Figure 15:**
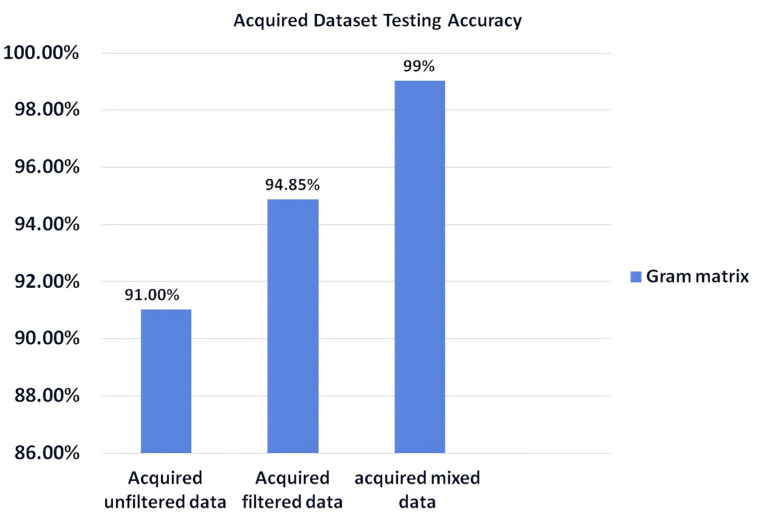
Testing accuracies for the three distinct datasets: raw PPG signals, filtered PPG signals, and mixed dataset.

The selected EfficientNetV2 B0 model integrated with LSTM was trained on three distinct datasets and subsequently tested to evaluate performance. The datasets included the raw PPG signals collected by our experimental setup, PPG signals filtered using the Savitzky-Golay filter, and a mixed dataset combining both filtered and unfiltered PPG signals. The training was conducted using the three different settings presented in Table 2, with 383 iterations per epoch and a total of 25 epochs, employing early stopping. Early stopping monitored the validation loss and halted training if no improvement was observed for 10 consecutive epochs to prevent overfitting. The testing accuracies, illustrated in Figure 15, show that the highest model performance—an accuracy of 99%—was achieved using the mixed dataset. This optimal result was obtained after fine-tuning the pre-trained model with Setting 2. In comparison, the other two settings yielded lower accuracies: 85% for Setting 1 and 94.6% for Setting 3, both using the same mixed dataset. Ultimately, this comparative analysis guided the development of the most effective training procedure for our real-time predictive system.

**Figure 16:**
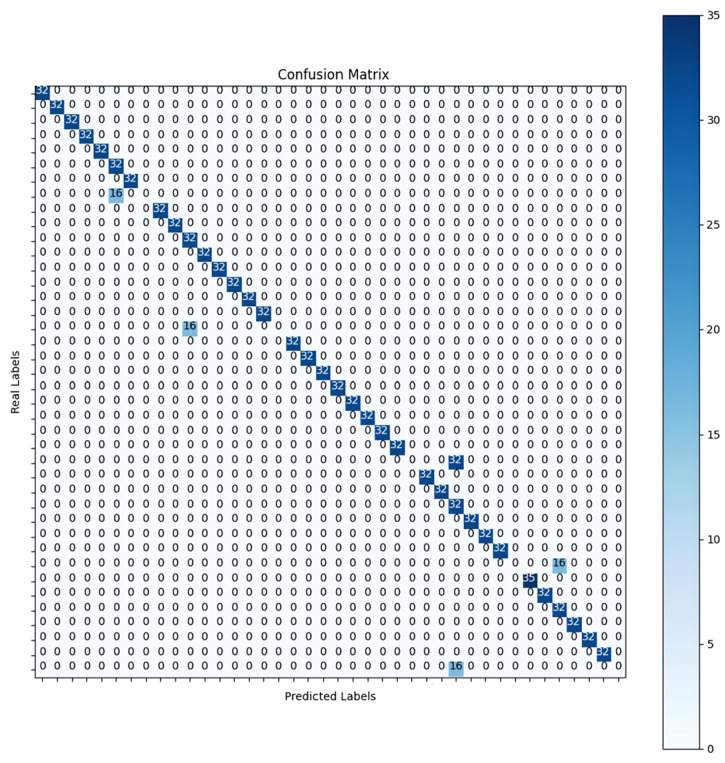
Confusion matrix for the EfficientNetV2 B0 model integrated with LSTM, evaluated on the mixed dataset.

Following testing, a confusion matrix was generated to comprehensively analyze the model’s performance across all 40 classes. Displayed in Figure 16, this matrix offers an in-depth comparison between the model’s predictions and the actual ground truth labels. Only five instances were misclassified, demonstrating the model’s high accuracy. The majority of the diagonal elements in the matrix represent true positives (TP), indicating the correct classification for each class. The five non-zero entries in the off-diagonal cells highlight the rare misclassifications, underscoring the model’s proficiency in distinguishing between classes. Overall, the confusion matrix confirms the model’s strong performance in accurate classification with minimal errors, validating its robustness and suitability for real-world applications.

After generating the confusion matrix, we developed a detailed classification report to further assess the model’s performance across all 40 classes. This report includes comprehensive metrics such as precision, recall, and F1 score for each class, as well as aggregated averages across all classes.

The analysis of the classification report revealed exceptional performance for most classes, with F1 scores, recall, and precision all achieving the maximum value of 1. This indicates that the model delivers highly accurate and precise classifications for nearly all classes, highlighting its robustness and effectiveness in distinguishing between different categories.

The weighted and macro average accuracies for our three distinct datasets are summarized in Table 3 and Table 4.

Overall, the model demonstrated exceptional performance on the mixed acquired dataset, achieving a weighted average accuracy of 99% for precision, recall, and F1 score. The macro average precision reached 99%, while the F1 score and recall both achieved values of 98%. These metrics underscore the model’s exceptional ability to generalize across all classes, highlighting its reliability and suitability for real-world applications. The results confirm the model’s effectiveness in accurately classifying instances across a diverse range of categories with high precision and recall.

The computational performance was evaluated by comparing EfficientNetV2 B0 integrated with LSTM across different hardware configurations (GPU, TPU, and CPU) and dataset types (filtered, unfiltered, and mixed), as presented in Table 5. For filtered datasets, the TPU demonstrated the fastest performance, with a training time of 1.5 h and a testing time of 10 min, significantly outperforming both the GPU and CPU. The GPU showed competitive performance, achieving a training time of 2.5 h, while the CPU lagged considerably, requiring 6 h for training.

For unfiltered datasets, the TPU again exhibited superior efficiency, reducing training time to 2 h, compared to 3 h on the GPU and 7 h on the CPU. For the mixed dataset, both the GPU and TPU achieved a good balance between speed and performance, with training times of 4 h and 2.5 h, respectively. Conversely, the CPU consistently displayed the slowest performance across all dataset types, highlighting its limitations in handling complex models and large datasets.

These results emphasize the importance of selecting the appropriate hardware configuration to optimize training and testing times, particularly when working with diverse and complex datasets. The TPU and GPU proved to be more efficient, especially in scenarios involving larger datasets and computationally intensive tasks.

### 4.2. Real-Time Identification

In a real-time identification scenario, the acquired signal is first preprocessed and converted into a 2D Gram matrix. This matrix is then used as input for the pretrained EfficientNetV2 B0 model integrated with LSTM, which was trained on the mixed dataset to generate predictions. Once identification is complete, the system plots the processed data and displays the predicted individual’s name using a Graphical User Interface (GUI) developed with Tkinter.

The GUI is designed with user-friendly buttons for initiating predictions and viewing results in a separate window. It also includes a restart button, enabling users to easily repeat the entire process if needed. This intuitive interface enhances the usability and accessibility of the real-time system, ensuring smooth operation and efficient interaction with the prediction results.

During the prediction phase, the system compares the acquired signal with the pre-trained data to produce accurate results. The prediction process typically takes about 25 to 30 s, providing a thorough analysis and precise outcomes. Importantly, all predictions made under these conditions are consistently accurate, demonstrating the system’s robustness.

**Figure 17:**
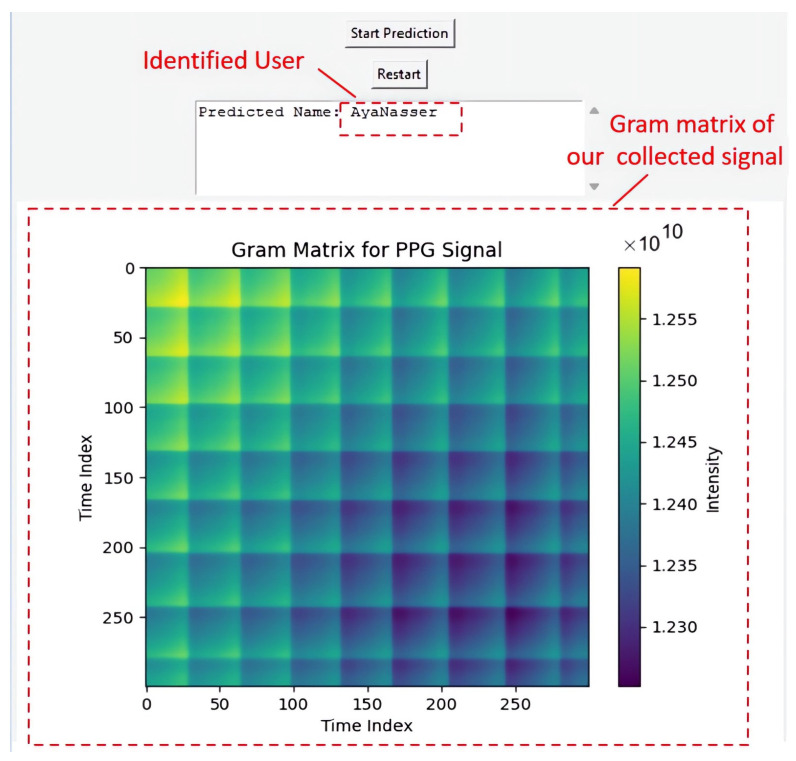
Example of real-time prediction, showcasing the predicted name and the corresponding preprocessed data for the acquired signal.

For a visualization of the prediction results, refer to Figure 17, which displays both the predicted name and the preprocessed data corresponding to the acquired signal.

These results highlight the effectiveness of utilizing PPG signals for biometric recognition and emphasize the efficiency of both the implemented system and the deep learning model.

## 5. Discussions

The growing integration of biometric systems for authentication has highlighted the need for advanced methods that ensure both high security and user convenience. Traditional biometric modalities, such as facial recognition, fingerprint scanning, and iris recognition, though widely adopted, face challenges in terms of susceptibility to spoofing, environmental variability, and user cooperation. In this context, photoplethysmography (PPG) signals emerge as a promising alternative, offering inherent advantages like liveness detection and difficulty in replication, which are critical for enhancing security in biometric systems.

Our study contributes to this evolving field by exploring the use of PPG signals, converted into a 2D Gram matrix representation, as a reliable source for biometric identification. By leveraging the EfficientNetV2 B0 model integrated with Long Short-Term Memory (LSTM), we demonstrate that deep learning techniques can effectively harness the rich, dynamic information encapsulated in PPG signals. Our approach not only achieves high accuracy but also addresses some of the limitations observed in previous studies.

### 5.1. Comparative Analysis

The results obtained from our experiments underscore the effectiveness of combining the EfficientNetV2 B0 model with LSTM in processing PPG signals for biometric recognition. The classification accuracy achieved, particularly when using a mixed dataset of filtered and unfiltered PPG signals, outperforms many existing methods reported in the literature. For instance, our model achieved a testing accuracy of 99% with minimal misclassifications, as evidenced by the confusion matrix. This performance is comparable to or exceeds that of previous studies utilizing PPG signals, such as the work by Labati et al., which also achieved 99% accuracy but employed different methodologies and preprocessing techniques.

In comparison with other biometric modalities like ECG or EMG signals, our method shows superior performance. For example, the study by Hanilci et al. achieved an accuracy of 88.57% using ECG signals, while Kim et al. reported a recognition performance of 97.2% with ECG data processed by deep CNNs. Our approach not only surpasses these results but also does so with the added benefit of using a more cost-effective and easier-to-acquire biometric trait—PPG signals.

### 5.2. Real-Time Application and Practical Implication

A significant advantage of our approach is its applicability in real-time scenarios. The system we developed, which involves real-time acquisition, preprocessing, and classification of PPG signals, demonstrates the practical feasibility of PPG-based biometric systems. The average prediction time of 25 to 30 s, combined with the system’s consistent accuracy, suggests that our approach is well-suited for deployment in environments requiring quick and reliable user authentication.

Moreover, the system’s design, featuring an intuitive Graphical User Interface (GUI), enhances usability and ensures that it can be easily integrated into existing biometric frameworks. The ability to capture and process PPG signals from any body part, facilitated by the simple and non-intrusive acquisition setup, further adds to the system’s versatility.

### 5.3. Challenges and Future Work

While our study demonstrates promising results, several challenges remain that warrant further investigation. One key issue is the variability in PPG signals due to factors such as skin tone, ambient lighting, and motion artifacts. Although our preprocessing techniques, including filtration and augmentation, mitigate some of these effects, there is still a need for more robust solutions that can handle a wider range of conditions.

Future research could explore the integration of additional physiological signals or multi-modal biometric systems to enhance accuracy and reliability further. Additionally, the development of more advanced data augmentation techniques and real-time noise reduction methods could improve system performance in diverse real-world settings.

### 5.4. Conclusions

In conclusion, our study highlights the potential of PPG signals as a reliable biometric modality for user identification. The combination of deep learning models like EfficientNetV2 B0 with LSTM provides a powerful tool for extracting meaningful features from PPG signals, resulting in high-accuracy biometric recognition. The successful implementation of a real-time identification system underscores the practicality of our approach, paving the way for future advancements in secure, efficient, and user-friendly biometric authentication systems.

## Figures and Tables

**Table 1:** Strengths and limitations of recent related studies.

Study	Signal Type	Advantages	Limitations	Performance Metrics
Li Zhang et al. (2018) [9]	PPG	High accuracy, effective deep learning models	Limited to fingertip data, small dataset size	High accuracy 93.6%
Hanilci et al. (2019) [10]	ECG	Efficient 2-D CNN architecture, fast processing	High computational complexity, long training times	Accuracy of 88.57%
M. Kim et al. (2019) [11]	ECG	Improved reliability, high accuracy	Increased computational complexity	Recognition performance of 97.2%
J. Kim et al. (2021) [12]	EMG	High gesture recognition performance	Data fusion complexity, overfitting risks	64.7% biometric performance
D. Labati et al. (2021) [13]	PPG	High accuracy, no fiducial points needed	Limited dataset scope, model adaptability	Accuracy 99%
M. Svetlakov et al. (2022) [14]	EEG	Subject-independent learning mechanism	Lower accuracy compared to other methods	Accuracy 64.7%
Bengie L. Ortiz et al. (2022) [15]	PPG	End-to-end learning, robust feature selection	Limited feature extraction techniques	Accuracy 97.36%
M. Ibrahim et al. (2023) [16]	PPG	Effective scalogram transformation, high accuracy	Complex feature fusion, high computational cost	Accuracy 95%
Caiyu Wu et al. (2022) [17]	PPG	Optimal 2-D representation, reduced computational demands	Overfitting risks, dependence on extensive datasets	Testing accuracy of 92.4%

**Table 2:** Hyperparameter settings for EfficientNetV2 B0 with LSTM.

Setting	Batch Size	Activation Function	Optimizer	Learning Rate	st	l
1	16	Softmax	SGD	0.01		
2	32	Relu	Adam	0.001		
3	64	Leaky Relu	RMSprop	0.0001		

**Table 3:** Weighted average accuracy of the classification report for our acquired datasets.

Datasets	F1 Score	Precision	Recall
Unfiltered Data	88%	88%	91%
Filtered Data	93%	91%	95%
Mixed Data	99%	99%	99%

**Table 4:** Macro average accuracy of the classification report for our acquired datasets.

Datasets	F1 Score	Precision	Recall
Unfiltered Data	87%	88%	89%
Filtered Data	86%	84%	88%
Mixed Data	98%	99%	98%

**Table 5:** Comparative computational performance of EfficientNetV2 B0 integrated with LSTM.

Dataset Type	Hardware CPU\GPU\TPU	Training Time (h)	Testing Time (s)	Memory Usage (GB)
Filtered	CPU\GPU\TPU	6\2.5\1.5	30\15\10	32\8\16
Unfiltered	CPU\GPU\TPU	7\3\2	40\20\12	32\10\16
Mixed Data	CPU\GPU\TPU	8\4\2.5	50\25\15	32\12\16

## Data Availability

Data are available upon request from the corresponding authors.
